# Oral cholera vaccine in cholera prevention and control, Malawi

**DOI:** 10.2471/BLT.17.207175

**Published:** 2018-04-23

**Authors:** Maurice M’bangombe, Lorenzo Pezzoli, Bruce Reeder, Storn Kabuluzi, Kelias Msyamboza, Humphreys Masuku, Bagrey Ngwira, Philippe Cavailler, Francesco Grandesso, Adriana Palomares, Namseon Beck, Allison Shaffer, Emily MacDonald, Mesfin Senbete, Justin Lessler, Sean M Moore, Andrew S Azman

**Affiliations:** a Ministry of Health, Lilongwe, Malawi.; b Department of Infections Hazard Management, World Health Organization, Geneva, Switzerland.; c College of Medicine, University of Saskatchewan, Saskatoon, Canada.; d Agence de Médecine Préventive, Paris, France.; e World Health Organization, Lilongwe, Malawi.; f The Malawi Polytechnic, Blantrye, Malawi.; g Epicentre, Paris, France.; h Médecins Sans Frontières France, Lilongwe, Malawi.; i International Vaccine Institute, Seoul, South Korea.; j Department of International Health, Johns Hopkins Bloomberg School of Public Health, Baltimore, United States of America (USA).; k Public Health Institute of Malawi, Lilongwe, Malawi.; l United Nations Children’s Fund, Lilongwe, Malawi.; m Department of Epidemiology, Johns Hopkins Bloomberg School of Public Health, 615 North Wolfe Street E6003 Baltimore, MD, USA.; n Department of Biological Sciences, University of Notre Dame, Notre Dame, USA.

## Abstract

**Problem:**

With limited global supplies of oral cholera vaccine, countries need to identify priority areas for vaccination while longer-term solutions, such as water and sanitation infrastructure, are being developed.

**Approach:**

In 2017, Malawi integrated oral cholera vaccine into its national cholera control plan. The process started with a desk review and analysis of previous surveillance and risk factor data. At a consultative meeting, researchers, national health and water officials and representatives from nongovernmental and international organizations reviewed the data and local epidemiological knowledge to determine priority districts for oral cholera vaccination. The final stage was preparation of an application to the global oral cholera vaccine stockpile for non-emergency use.

**Local setting:**

Malawi collects annual data on cholera and most districts have reported cases at least once since the 1970s.

**Relevant changes:**

The government’s application for 3.2 million doses of vaccine to be provided over 20 months in 12 districts was accepted in April 2017. By April 2018, over 1 million doses had been administered in five districts. Continuing surveillance in districts showed that cholera outbreaks were notably absent in vaccinated high-risk areas, despite a national outbreak in 2017–2018.

**Lessons learnt:**

Augmenting advanced mapping techniques with local information helped us extend priority areas beyond those identified as high-risk based on cholera incidence reported at the district level. Involvement of the water, sanitation and hygiene sectors is key to ensuring that short-term gains from cholera vaccine are backed by longer-term progress in reducing cholera transmission.

## Résumé

**Problème:**

En raison de stocks mondiaux réduits de vaccins anticholériques oraux, les pays doivent identifier les zones prioritaires en matière de vaccination tout en développant des solutions à plus long terme, telles que des infrastructures d'alimentation en eau et d'assainissement.

**Approche:**

En 2017, le Malawi a intégré la vaccination anticholérique orale dans son plan national de lutte contre le choléra. Le processus a commencé par un examen documentaire et une analyse des données disponibles concernant la surveillance et les facteurs de risque. Lors d'une réunion consultative, des chercheurs, les responsables nationaux des secteurs de la santé et de l'eau et les représentants d'organisations non gouvernementales et internationales ont examiné les données et les connaissances épidémiologiques locales pour déterminer les districts où devait être pratiquée en priorité la vaccination anticholérique orale. L'étape finale a consisté à préparer une demande relative au stock mondial de vaccins anticholériques oraux destinés à être utilisés en dehors des situations d'urgence.

**Environnement local:**

Le Malawi collecte des données annuelles sur le choléra et la plupart des districts ont signalé des cas à au moins une reprise depuis les années 1970.

**Changements significatifs:**

La demande du gouvernement visant la fourniture de 3,2 millions de doses de vaccins pendant 20 mois dans 12 districts a été acceptée en avril 2017. En avril 2018, plus d'un million de doses ont été distribuées dans cinq districts. Une surveillance continue au sein des districts a révélé un fait frappant, à savoir que les zones à risque épidémiologique élevé ayant fait l'objet d'une vaccination n'ont pas été touchées par des épidémies de choléra, malgré une épidémie nationale en 2017-2018.

**Leçons tirées:**

L'association de techniques avancées de cartographie à des informations locales nous a permis d'élargir les zones prioritaires au-delà de celles identifiées comme présentant un risque épidémiologique élevé en fonction de l'incidence du choléra signalée au niveau des districts. La mobilisation des secteurs de l'eau, de l'assainissement et de l'hygiène est essentielle pour garantir que les avantages à court terme des vaccins anticholériques sont confirmés par un recul à plus long terme de la transmission du choléra.

## Resumen

**Problema:**

Con suministros mundiales limitados de la vacuna oral contra el cólera, los países deben identificar las áreas prioritarias para la vacunación mientras se desarrollan soluciones a más largo plazo, como las infraestructuras de agua y el saneamiento.

**Enfoque:**

En 2017, Malawi integró la vacuna oral contra el cólera en su plan nacional para el control del cólera. El proceso empezó con un estudio preliminar y un análisis de los datos previos de vigilancia y factores de riesgo. En una reunión consultiva, investigadores, funcionarios nacionales de salud y agua y representantes de organizaciones no gubernamentales e internacionales revisaron los datos y el conocimiento epidemiológico local para determinar los distritos prioritarios para la vacunación oral contra el cólera. La etapa final fue la preparación de una aplicación a la reserva global de la vacuna oral contra el cólera para usos de no emergencia.

**Marco regional:**

Malawi recopila datos anuales sobre el cólera y la mayoría de los distritos han registrado casos al menos una vez desde los años setenta.

**Cambios importantes:**

La solicitud del gobierno de 3,2 millones de dosis de vacunas para suministrarlas a lo largo de 20 meses en 12 distritos se aceptó en abril de 2017. En abril de 2018, se habían administrado más de 1 millón de dosis en cinco distritos. La vigilancia constante en los distritos mostró que los brotes de cólera estuvieron notablemente ausentes en las áreas vacunadas de alto riesgo, a pesar de un brote nacional en 2017-2018.

**Lecciones aprendidas:**

El aumento de las técnicas avanzadas de mapeo con información local ayudó a ampliar las áreas prioritarias más allá de las identificadas como de alto riesgo según la incidencia del cólera registrada a nivel de distrito. La participación de los sectores del agua, el saneamiento y la higiene es clave para garantizar que las victorias a corto plazo de la vacuna contra el cólera estén respaldadas por un progreso a más largo plazo en la reducción de la transmisión del cólera.

## ملخص

**المشكلة:**

مع الشح العالمي في إمدادات تطعيم الكوليرا عبر الفم، تحتاج البلدان إلى تحديد المناطق التي تستحق الأولوية في تقديم التطعيمات في الوقت الذي يتم فيه تطوير حلول أطول مدى، مثل بناء البنية التحتية للمياه والصرف الصحي.

**الأسلوب:**

قامت ملاوي في عام 2017 بإدماج تطعيم الكوليرا عبر الفم في خطتها الوطنية للسيطرة على مرض الكوليرا. وبدأت العملية باستعراض مستندي وتحليل لبيانات الرصد السابقة وعوامل الخطورة. وفي اجتماع استشاري، قام الباحثون، والمسؤولون المختصون بشؤون الصحة والمياه على مستوى البلاد، وممثلون عن مؤسسات دولية وغير حكومية بمراجعة البيانات والمعرفة المحلية عن الأوبئة لتحديد المقاطعات المستحقة للأولوية في التعامل مع تطعيم الكوليرا عبر الفم. وكانت المرحلة الأخيرة تتمثل في تحضير طلب للاستفادة من المخزون العالمي لتطعيم الكوليرا عبر الفم لاستخدامه في غير حالات الطوارئ.

**المواقع المحلية:**

تقوم ملاوي بتجميع البيانات السنوية حول الكوليرا، وقامت أغلب المقاطعات بتقديم بلاغات عن الحالات مرة واحدة على الأقل منذ سبعينيات القرن العشرين.

**التغييرات ذات الصلة:**

تم قبول طلب الحكومة للحصول على 3.2 مليون جرعة من التطعيم ليتم تقديمها على مدار 20 شهرًا في 12 مقاطعة في شهر أبريل/نيسان من عام 2017. وبحلول شهر أبريل/نيسان من عام 2018، تم تقديم أكثر من مليون جرعة في خمس مقاطعات. وأظهرت عمليات الرصد المستمرة في المقاطعات أن حالات تفشي الكوليرا قد اختفت بشكل ملحوظ في المناطق المحصنة المعرضة لمخاطر عالية، على الرغم من تفشي المرض على مستوى البلاد في عام 2017-2018.

**الدروس المستفادة:**

لقد استفدنا من توظيف المعلومات المحلية لاستكمال أساليب المطابقة المتقدمة وذلك لمد نطاق مناطق الأولوية بما يتعدى تلك المناطق التي تم تحديدها باعتبارها مناطق عالية الخطورة من واقع تفشي الكوليرا التي يتم الإبلاغ على مستوى المقاطعات. وتلعب مشاركة قطاعات المياه والصرف الصحي والصحة دورًا رئيسيًا في ضمان تدعيم المكاسب قصيرة المدى من التطعيم بالكوليرا بفعل التقدم المحقق على المدى الأبعد في مجال الحد من انتقال الكوليرا.

## 摘要

**问题:**

由于全球口服霍乱疫苗的供应有限，各国需要确定接种疫苗的优先区域，而长期的解决方案（例如饮水和卫生基础设施）尚在发展中。

**方法:**

2017 年，马拉维将口服霍乱疫苗纳入其全国霍乱控制计划。进程开始于书面材料审查和对之前监控与风险因素数据的分析。在一次协商会议上，研究人员、全国健康与水资源官员和来自非政府组织与国际组织的代表评估了数据以及对当地流行病的认识，决定了口服霍乱疫苗的优先地区。最终阶段是非紧急用途下为全球口服霍乱疫苗储备申请做准备。

**当地状况:**

马拉维收集了霍乱的年度数据，自 20 世纪 70 年代以来，绝大部分地区都汇报过至少一次病例。

**相关变化:**

截至 2017 年 4 月，政府在 20 多个月内为 12 个地区提供 320 万支疫苗的申请被批准。截至 2018 年 4 月，超过 100 万支疫苗在 5 个地区被使用。各地区的持续监控显示，尽管在 2017 年至 2018 年出现了一次全国性的霍乱爆发，但在注射疫苗的高风险地区霍乱爆发的情况明显消失。

**经验教训:**

强化先进的测绘技术并结合当地信息，有助于我们基于地区层面有关霍乱病例的汇报来确定除霍乱爆发的高风险地区之外的优先区域。饮水、环境卫生和卫生部门的参与对于确保霍乱疫苗的短期收益能够得到减少霍乱传播的长期过程支持非常关键。****

## Резюме

**Проблема:**

В условиях ограниченного глобального обеспечения пероральной вакциной против холеры странам необходимо определить приоритетные области для вакцинации одновременно с разработкой долгосрочных решений, таких как объекты инфраструктуры водоснабжения и санитарии.

**Подход:**

В 2017 году Малави включила пероральную вакцину против холеры в свой национальный план борьбы с этим заболеванием. Процесс начался с камеральной проверки и анализа ранее полученных данных эпиднадзора и информации о факторах риска. На консультативном совещании исследователи, должностные лица в области общественного здравоохранения и водных ресурсов и представители неправительственных и международных организаций изучили данные о местной эпидемиологической обстановке и факторах риска для определения приоритетных районов для вакцинации против холеры. Заключительным этапом стало создание заявки на получение из глобальных резервных запасов пероральной вакцины против холеры для проведения плановой вакцинации.

**Местные условия:**

Малави собирает ежегодные данные о холере, и большинство районов сообщили по крайней мере об одном случае заболевания с 1970-х годов.

**Осуществленные перемены:**

В апреле 2017 года была принята заявка правительства на 3,2 миллиона доз вакцины, которые должны быть предоставлены в течение 20 месяцев в 12 районах. К апрелю 2018 года в пяти районах было введено более 1 миллиона доз вакцины. Действующий на местах эпиднадзор показал, что в районах повышенного риска, в которых была проведена вакцинация, отсутствовали вспышки холеры, несмотря на общенациональную вспышку в 2017–2018 годах.

**Выводы:**

Совместное использование передовых методов картирования и информации о местной эпидемиологической обстановке помогло авторам расширить приоритетные области, помимо тех, которые были определены как районы повышенного риска по заболеваемости холерой, зарегистрированному на районном уровне. Участие секторов водоснабжения, санитарии и гигиены имеет ключевое значение для обеспечения того, чтобы краткосрочные выгоды от вакцинации против холеры подкреплялись долгосрочным прогрессом в сокращении передачи этого заболевания.****

## Introduction

Cholera continues to be a global public health threat, with 150 000 cases per year reported from sub-Saharan Africa.^1^ While universal access to safe water and sanitation is the ultimate solution, the development of such infrastructure and associated behaviour change may take years at the current pace.^2^ Relieving the burden of cholera today requires both short-term and long-term approaches.

Oral cholera vaccine, which protects for at least three years,^3^ can play an important role in short-term risk reduction, complementing long-term water and sanitation interventions. More than 20 000 000 doses of the vaccine have been used across the world, primarily during emergencies.^4^ In the light of increased global availability of the vaccine, in 2015 the global cholera vaccine stockpile^4^^,^^5^ was extended for use in areas experiencing recurrent cholera outbreaks. Emergency vaccination campaigns, including campaigns in Malawi,^6^^,^^7^ have largely been conducted with the support of nongovernmental organizations (NGOs), with little integration into local health systems. Vaccine use in areas with recurrent cholera transmission provides an opportunity for comprehensive cholera prevention integrated with government health and infrastructure programmes.

The Malawi national cholera prevention and control plan, developed in 2016–2017, took a comprehensive approach that includes enhanced leadership, disease surveillance, laboratory support, clinical care, cholera vaccination, provision of safe water and sanitation, and social and behavioural changes. To ensure the best use of the vaccine, which is typically delivered to the whole population (≥  1 years old) in mass campaigns,^4^ the plan called for the elaboration and implementation of a national cholera vaccine deployment plan in areas with regular cholera transmission. Here we describe the process of synthesizing historical data and local knowledge across sectors to plan this first multi-year non-emergency use of the stockpile.

## Local setting

**Fig. 1:**
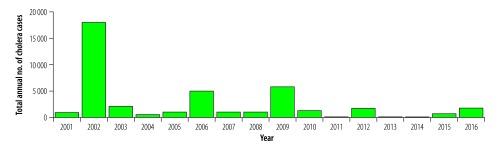
Total annual number of suspected cholera cases in Malawi, 2001–2016

**Fig. 2:**
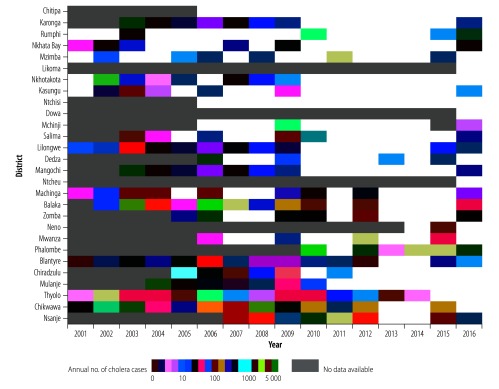
Annual number of suspected cholera cases by district of Malawi, 2001–2016

We collated data on cases of cholera in Malawi from 2001–2016, aggregated across different spatial and temporal scales. Based on annual district-level data, over this period 41 316 suspected cholera cases were reported in Malawi (population 12 978 432 in the 2008 census; Fig. 1), with 23 out of 28 districts ever reporting cases (Fig. 2). Cases were reported almost every year. Seven districts were responsible for 33 276 (80.5%) of the 41 316 cumulative cases reported, with six districts reporting cholera cases for more than half of the years (Table 1). These district-level analyses, which give equal importance to cases reported in 2001 and 2016, obscured important heterogeneities and trends in incidence.

**Table 1:** Overview of the national cholera vaccine planning process and progress in Malawi

District	District-level data review (2001–2016)					Subdistrict-level analyses (2010–2016)		Decisions			Implementation and surveillance		
	Ever reported cholera	Responsible for first 80% of cases^a^	Reported for most years	Moderate-to-high incidence^b^	Low coefficient of variation^c^	High-incidence subdistricts^d^	Moderate-to-high incidence subdistricts^d^	Contains key at-risk populations^e^	Prone to flooding	Priority district^f^	No. of vaccine doses planned	No. of vaccine doses delivered^g^	No. of cholera cases^g^
Balaka	Y	Y	Y	Y	N	N	N	N	N	N	0	0	0
Blantyre	Y	Y	Y	Y	N	N	Y	Y	Y	**Y**	364 816	0	1
Chikwawa	Y	Y	Y	Y	Y	Y	Y	Y	Y	**Y**	412 138	486 510	1
Chiradzulu	Y	N	N	N	N	N	N	N	N	N	0	0	0
Chitipa	N	N	N	N	N	N	N	N	N	N	0	0	0
Dedza	Y	N	N	N	N	N	N	N	N	N	0	0	31
Dowa	N	N	N	N	N	N	N	Y	N	**Y**	57 000	89 500	5
Karonga	Y	N	N	Y	N	N	Y	Y	Y	**Y**	174 648	216 000	347
Kasungu	Y	Y	N	Y	N	N	N	N	Y	N	0	0	1
Likoma	N	N	N	N	N	N	N	Y	N	N	0	0	13
Lilongwe	Y	Y	Y	Y	N	N	N	Y	Y	**Y**	228 000	0	348
Machinga	Y	Y	Y	Y	N	Y	Y	Y	N	**Y**	340 000	0	0
Mangochi	Y	N	N	Y	N	N	N	Y	N	**Y**	176 232	0	0
Mchinji	Y	N	N	N	N	N	N	N	N	N	0	0	0
Mulanje	Y	N	N	N	Y	N	N	N	N	N	0	0	4
Mwanza	Y	N	N	Y	N	N	N	N	N	N	0	0	0
Mzimba	Y	N	N	N	N	N	Y	N	N	N	0	0	0
Neno	Y	N	N	N	N	N	N	N	N	N	0	0	0
Nkhata Bay	Y	N	N	Y	Y	N	Y	Y	N	**Y**	236 618	0	20
Nkhotakota	Y	N	N	Y	N	N	N	N	N	N	0	0	0
Nsanje	Y	Y	N	Y	Y	Y	Y	Y	Y	**Y**	340 000	40 000	6
Ntcheu	N	N	N	N	N	N	N	N	N	N	0	0	0
Ntchisi	N	N	N	N	N	N	N	N	N	N	0	0	0
Phalombe	Y	N	N	N	N	N	Y	Y	N	**Y**	351 102	0	0
Rumphi	Y	N	N	N	N	N	Y	Y	N	N	0	0	13
Salima	Y	N	N	Y	N	N	Y	Y	Y	**Y**	216 618	217 064	99
Thyolo	Y	N	Y	N	Y	N	Y	N	N	N	0	0	0
Zomba	Y	N	N	Y	Y	Y	Y	Y	N	**Y**	334 578	0	0

Y: yes; N: no.

^a^ Districts that contributed to the first 80% of total cases from 2001–2016 when ordered from highest to lowest number of total cases.

^b^ Moderate-to-high mean annual incidence was defined as > 1 cholera case per 10 000 population.

^c^ Lowest 25th percentile

^d^ High-incidence subdistricts were defined as those with ≥ 10% of the population or ≥ 100 000 people living in an area with an annual cholera incidence > 1 per 1000 population; moderate incidence subdistricts were those with mean annual incidence > 1 per 10 000 population.

^e^ Key populations at-risk of cholera were defined as fisherman, refugees and internally displaced persons.

^f^ Districts designated as priority districts in the oral cholera vaccine plan are shown in bold type.

^g^ Data are for the period 1 October 2017 to 8 April 2018.

Notes: The number of doses delivered was sometimes above what was planned because there were changes in the population at-risk particularly due to outbreaks. Karonga and Salima campaigns were accelerated due to outbreaks that started before vaccination. Due to the geographically widespread outbreak in Lilongwe, the target population was expanded for a planned campaign in April to May 2018.

## Approach

**Fig. 3:**
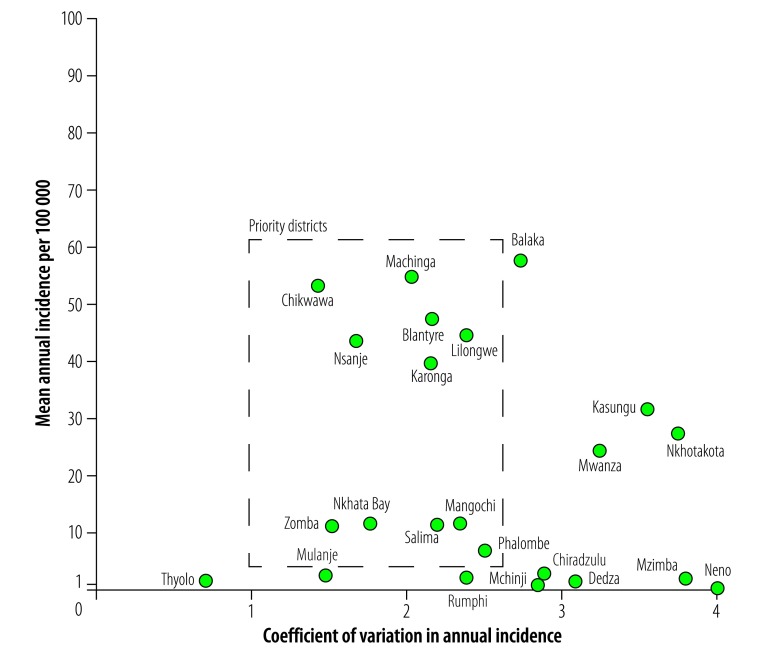
District-level annual incidence of suspected cholera versus coefficient of variation in annual incidence in Malawi, 2001–2016

Note: Districts selected as priority for oral cholera vaccination are shown within the dotted boundary. Dowa district is not included as no cases were reported in this district since 2001.

The process started in early 2016 with a desk review and analysis of previous surveillance and risk factor data. To prioritize districts for vaccination we focused on three measures of burden for each district: (i) mean annual incidence of cholera (cholera in this paper refers to suspected cholera as cases were not systematically confirmed); (ii) its coefficient of variation (capturing the year-to-year variability in incidence); and (iii) the number of years of reporting cholera. Using Bayesian mapping techniques we combined cholera case reports from 2010–2016 and data on population density^8^ and on access to water and sanitation^9^ to map the average annual cholera incidence in areas of 20 km × 20 km and to classify subdistricts according to different incidence thresholds.^1^ Based on the mapping analyses, we found that 437 023 people (95% credible interval, CrI: 96 154‒1 604 563) from four districts lived in high-incidence subdistricts (≥ 10% of the population or ≥ 100 000 people living in an area with annual cholera incidence > 1 per 1000 population). A total of 2 429 009 people (95% CrI: 648 642‒4 872 006) from 12 districts lived in moderate-to-high incidence subdistricts where the mean annual incidence exceeded 1 per 10 000 population. The majority of subdistricts with high or moderate-to-high incidence were located in three main areas: lower Shire River basin, Lake Chilwa area and northern Lake Malawi area (Fig. 3). Data from these areas were similar to but not in complete agreement with the results of the annual district-level analyses (Table 1).

From 28 February 2017 to 2 March 2017, stakeholders met to validate the Malawi national cholera prevention and control plan and to propose an integrated national oral cholera vaccine plan, to submit a request to the global stockpile. National and district-level health ministry officials joined experts from NGOs, international organizations and academia, to review the epidemiological data. This process was supplemented with contextual data (such as access to water, sanitation and hygiene) and with qualitative information presented by district health leaders representing each of Malawi’s three regions. These presentations highlighted specific populations (such as fishermen) that were disproportionally affected by cholera and which routine surveillance data did not capture. Stakeholders worked in groups to prioritize districts based on the raw and summary data. Each group presented the rationale for their prioritization and were offered an opportunity to adjust these according to comments from the whole group. The key reasons for selecting high-priority districts were similar across groups and included: historic cholera incidence (focused on 2011–2016); frequency of cholera reports; local water and sanitation conditions; presence of difficult environmental or social conditions for improving water and sanitation infrastructure; a shared border with Mozambique; and the presence of key populations such as fishermen and refugees.

**Fig. 4:**
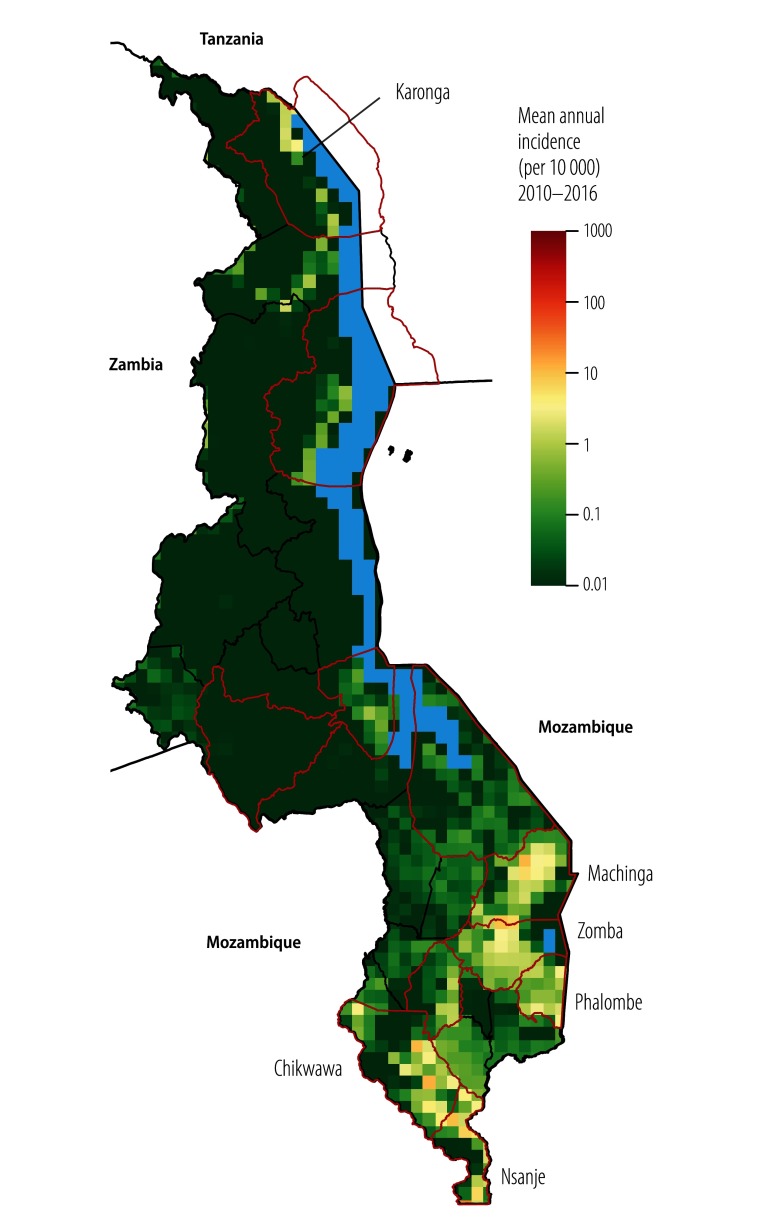
Mean annual incidence of suspected cholera in Malawi, 2010–2016

Note: Districts selected as priority for oral cholera vaccination are highlighted with red outlines.

After the two-day deliberation, we compiled the district rankings from each stakeholder group, with a clear consensus on five high-priority districts (Chikwawa, Machinga, Nsanje, Phalombe and Zomba). Another four districts (Blantyre, Karonga, Lilongwe and Salima) were high-priority for half or more of the groups. Three additional districts (Dowa, Mangochi and Nkhatabay) were high-priority for at least one group and medium priority for the rest (Table 1). The stakeholders agreed that these 12 districts would move to the final planning stage, where priority subpopulations would be identified. All priority districts except Dowa, home to a large refugee camp, frequently report cases (that is, low coefficient of variation in Fig. 4) with relatively high mean annual incidence and were identified as moderate-to-high incidence districts in the data review exercises conducted before the meeting (Table 1).

Next, a health ministry-led team took the recommendations from the stakeholder meeting and began drafting a national oral cholera vaccine plan. Within each priority district, district health officials were asked to select priority areas for vaccination, considering three components: (i) history of confirmed cholera; (ii) water and sanitation indicators; and (iii) presence of high-risk populations such as fishermen and displaced people. No limit was placed on the number of potential high-priority people proposed for each district, but the combined population of more than 9 million from all 12 districts would likely be too large as a national allocation from current global vaccine supplies.

In the final stage, in consultation with United Nations Children’s Fund, the Ministry of Agriculture, Irrigation and Water Development and others, the health ministry drafted an application for 3.2 million doses of vaccine to be provided in six phases over 20 months in 12 districts. The vaccine would be targeted on all people aged 1 year and older, including pregnant women, and delivered through mass campaigns centred on health ministry facilities. Vaccines would be administered by the community health workers who were typically involved in routine immunization activities. Along with each campaign, several partners, including NGOs and national and international academic institutions, agreed to assist with monitoring and evaluation. These activities included coverage surveys, longer-term impact assessments and enhanced surveillance, all designed to help improve future oral cholera vaccine programmes and to better understand how oral cholera vaccine may affect the burden of disease in the medium to long term. The application was reviewed by the Global Taskforce for Cholera Control and accepted in April 2017.

## Relevant changes

Limited global supplies of oral cholera vaccine and competing emergency response needs led to a slow initial start to implementation of the plan. Between June 2017 and April 2018, 1 049 074 doses of vaccine were administered via campaigns in five districts (Chikwawa, Dowa, Karonga, Nsanje and Salima; Table 1). Another 1 million vaccine doses, were expected to be delivered between April and May 2018 for Lilongwe district. No formal analyses of vaccine coverage were made, but surveys showed that coverage (number of doses delivered divided by number of people targeted) was generally high in the campaigns: > 70% for two doses. It is too early to assess the impact of cholera vaccine deployments in Malawi. However, a cholera outbreak that affected all regions of Malawi from October 2017 to March 2018 resulted in few or no cases in Nsanje, Chikwawa and the Lake Chilwa area, all which have had almost annual outbreaks with moderate-to-high incidence since 2001, but had been recently vaccinated (Table 1).^6^^,^^7^

## Lessons learnt

A review of historical data supplemented by the experience of local public health professionals was an efficient method to identify priority populations and plan for short-term cholera prevention activities, while yielding key data to help benchmark progress in eliminating cholera (Box 1). Years of cholera prevention work lie ahead, but these exercises set a new standard for making data-driven decisions within the country and provide valuable lessons for other countries moving forward with national cholera prevention efforts.

Box 1 **Summary of main lessons learnt**

- Collation of historical data on cholera incidence was an opportunity to discuss cholera planning with each of the district governments and provided a common baseline from which to discuss priority areas.
- Augmenting detailed analyses with local information on key populations affected by cholera helped us refine priority areas and in some cases to extend priority areas beyond those identified as high-risk based solely on recent cholera incidence.
- Although vaccines are usually deployed through the health sector, involvement of the water, sanitation and hygiene sectors is key to ensuring that short-term gains from cholera vaccine are backed by longer-term progress in reducing cholera transmission.

Oral cholera vaccine can be planned and deployed relatively quickly compared with sustainable water and sanitation interventions. Therefore, if water and sanitation needs are not met during the coming few years, revaccination of part, or all the population, may be required to sustain the anticipated reductions in cholera. To truly minimize cholera risk, along with numerous other health and social benefits, every effort must be made to achieve sustainable water and sanitation for all, even if this takes several vaccination cycles.

Outbreaks of cholera during the roll-out of vaccination are likely in countries like Malawi. While the plan did not explicitly address how the health ministry would adapt to urgent needs in the face of an outbreak, flexibility has been key to using the vaccine reactively and delaying vaccination in other less urgent locations. Future plans should make clear how these competing priorities will be balanced.

Maintaining sensitive and specific surveillance for cholera (including appropriate use of rapid diagnostic tests and regular training of district health officials) in the upcoming years will be key to measuring the impact of the comprehensive cholera control programme and to guide timely and appropriate responses to cholera outbreaks (Box 1).
